# Impact of health literacy and subjective happiness in pregnancy on neonatal anthropometry: a cohort study

**DOI:** 10.1007/s44192-025-00192-8

**Published:** 2025-04-24

**Authors:** Samira Silakhori, Safa Mousavi, Sadra Sarandili, Mojgan Rahmanian

**Affiliations:** 1 Abnormal Uterine Bleeding Research Center, Semnan University of Medical Sciences, Semnan, Iran; 2 California State University, Fresno, CA USA; 3 Department of Health Sciences, Curtin Medical School, Curtin University, Perth, WA Australia

**Keywords:** Pregnancy, Maternal health literacy, Anthropometry, Neonates

## Abstract

**Background:**

Maternal health literacy (MHL) and happiness can significantly impact pregnancy outcomes (POs) and neonatal health. This study primarily aimed to assess the level of maternal health literacy. Additionally, we sought to determine how maternal health literacy, happiness, socio-demographics, and pregnancy outcomes influence neonatal anthropometrics, thereby providing a more comprehensive understanding of the determinants of maternal and neonatal health.

**Methods:**

In a 2-year cohort study on 591 pregnant women with normal pregnancies, we recorded the maternal socio-demographic and obstetric factors. Moreover, the Maternal Health Literacy and Pregnancy Outcomes Questionnaire (MHLAPQ) and Happiness Questionnaire were used to gather health-related information. After delivery, the neonatal anthropometric measurements, including body weight (BW), supine length (SL), and head circumference (HC), were recorded at birth.

**Results:**

Among the 591 participants, 338 (57.2%) were in the 20–29-year age group. The mean maternal health literacy (MHL) score was 57.34 ± 8.67. MHL scores were associated with maternal and paternal education and occupation, as well as a history of miscarriage. Pregnancy outcomes were linked to maternal and paternal education and employment status, while higher happiness scores were found among housewives. BW showed significant differences based on maternal occupation and a history of stillbirth, while SL varied significantly with maternal occupation. Positive correlations were identified between BW and MHL, as well as between PO, MHL, and maternal happiness scores.

**Conclusion:**

In summary, our results revealed that MHL and happiness are important factors in improving the POs and neonatal health. In addition, maternal and paternal education and occupation were found to affect the MHL scores.

**Supplementary Information:**

The online version of this article (10.1007/s44192-025-00192-8) contains supplementary material, which is available to authorized users.

## Introduction

Anthropometric indicators at birth play a pivotal role as critical health markers frequently employed to evaluate the overall health and chances of survival for neonates [1]. Low birth weight, a crucial developmental indicator, is defined as a weight below 2500 g, comprising approximately 15–20% of all global births [2]. In developing countries, the prevalence of underweight infants ranges from 5 to 7%, while in developed nations, it rises to 19% [2]. Notably, studies in Iran have reported a prevalence between 8 and 11% [3, 4].

Fetal growth and weight, along with height and head circumference (HC) at birth, are influenced by a multitude of factors during and before pregnancy. Maternal characteristics significantly shape these anthropometric indicators, with genetic factors, preterm labor, multiple pregnancies, maternal illnesses during pregnancy, drug and tobacco use history, maternal age, and maternal overweight and obesity emerging as notable influencers [5]. Genetic, social, and biological factors, as well as those affecting the mother’s health, also influence fetal development [6]. The mother’s lifestyle choices, including smoking, pose a threat to fetal health due to the presence of nicotine and other harmful compounds [7].

Among these various determinants, maternal health literacy (MHL) has emerged as a crucial factor influencing both pregnancy outcomes (POs) and neonatal health. MHL encompasses cognitive and social skills that determine a mother’s ability to obtain, comprehend, and use health information to make appropriate decisions regarding prenatal care, nutrition, and avoidance of harmful substances [8]. Adequate MHL can improve adherence to medical advice, prompt utilization of prenatal care services, and better understanding of fetal health needs, ultimately reducing the risk of complications and improving neonatal anthropometric parameters such as birth weight (BW) and length. In contrast, insufficient MHL may lead to delayed recognition of pregnancy-related problems, poor dietary choices, and lower utilization of preventive measures, thereby negatively affecting fetal growth and neonatal well-being [9].

Additionally, maternal emotional well-being—particularly happiness—plays an integral role in influencing the course of pregnancy and fetal development. Happiness, as a positive emotional state, can buffer against stress, anxiety, and depression, all of which have been associated with adverse pregnancy outcomes, including preterm delivery and lower birth weight [10]. Pregnant women experiencing higher levels of happiness are more likely to engage in health-promoting behaviors, maintain better nutrition, and adhere to medical advice, thereby fostering an environment conducive to optimal fetal growth [11, 12]. Conversely, unhappiness or significant psychosocial stress can contribute to unfavorable pregnancy outcomes and suboptimal neonatal anthropometric measurements [10]. Understanding the interplay between maternal happiness and neonatal health is therefore essential for designing comprehensive prenatal care strategies that address both physical and psychosocial needs.

The relationship between fetal growth indices—such as BW, HC, and supine length (SL)—and MHL, pregnancy outcome, happiness, and maternal socio-demographic characteristics remains relatively unexplored. The primary objective of this study was to assess the level of maternal health literacy. Secondary objectives included examining the association between maternal health literacy and key neonatal anthropometric parameters at birth, and investigating the relationship between maternal happiness, pregnancy outcomes, and socio-demographic factors with neonatal anthropometric measurements. By clarifying these aims, this study seeks to fill critical gaps in the health literature and provide evidence to guide interventions aimed at improving maternal and neonatal health.

## Materials and methods

### Study design

This cohort study was conducted at Amir Al-Momenin Hospital, affiliated with Semnan University of Medical Sciences in Semnan, Iran, from May 25th, 2017, to January 31st, 2019. Participants were pregnant women referred to the hospital, identified through routine referrals and healthcare provider recommendations.

#### Primary outcome and sample size calculation

The primary outcome of this study was the Maternal Health Literacy (MHL) level, assessed by the Maternal Health Literacy and Pregnancy Outcomes Questionnaire (MHLAPQ). To determine the sample size, we applied a single-sample proportion formula with a 95% confidence level, a 5% margin of error, and a 10% allowance for attrition, using an estimated proportion of approximately 30% insufficient MHL based on regional data. The sample size calculation was performed using PASS software (NCSS, LLC, Kaysville, UT, USA) [9]:

$${\text{n}} = {\text{Z}}^{2} \;*\;{\text{p}}\;*\;{\text{q}}/{\text{E}}^{2} .$$

This yielded a minimum required sample size of 323 participants. Considering the nature of the study (a 2-year cohort design) and potential sample loss, 780 subjects were enrolled in this study. Finally, 650 pregnant women meeting the inclusion and exclusion criteria were recruited from a single center in Semnan, Iran, during the study period.

#### Secondary outcomes

Secondary outcomes included maternal happiness scores (assessed using the Happiness Questionnaire), pregnancy outcome (PO) scores (from the MHLAPQ), and neonatal anthropometric measurements (birth weight, supine length, and head circumference).

**Fig. 1:**
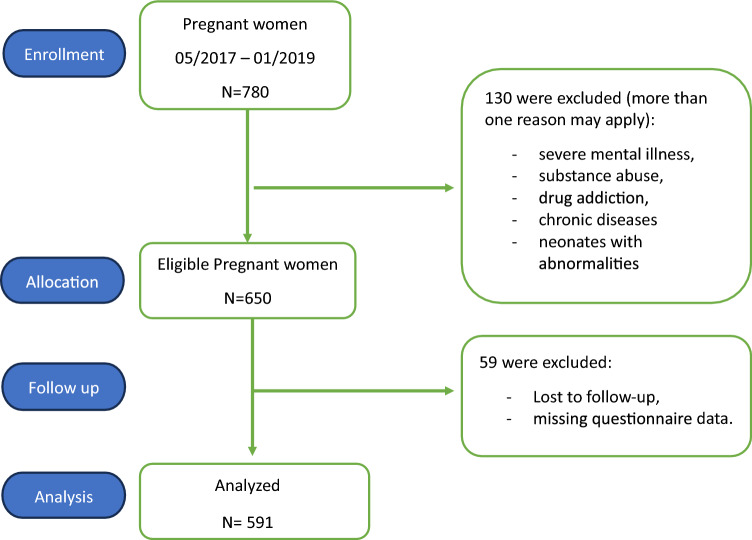
The study selection process

All participants were provided with a comprehensive explanation of the study’s purpose and procedures and gave informed consent. After demographic data collection, they completed the MHL questionnaire. Participants were reminded of their right to withdraw at any time. Maternal and neonatal data were recorded after delivery. Following the application of inclusion and exclusion criteria, data from 591 women were included in the final analysis. Figure 1 illustrates the consort diagram outlining cohort inclusion and exclusion criteria (Fig. 1).

### Participant selection procedure

The process of participant selection for this research entailed the establishment and application of specific inclusion and exclusion criteria. These criteria were formulated with the aim of securing a comprehensive representation of neonates and their mothers while taking into consideration factors that could potentially impact the relationship under investigation. The inclusion criteria encompassed conditions such as a healthy pregnancy and Iranian nationality, regardless of gestational age (GA), absence of severe mental illness, substance abuse, or drug addiction, as well as a lack of significant stress experiences in the past year, including chronic diseases, immigration, or the loss of a close relative, along with a demonstrated willingness to cooperate in the study. Conversely, the exclusion criteria were designed to mitigate potential confounding factors that could influence the outcomes under scrutiny, leading to the exclusion of individuals with a history of severe mental illness, substance abuse, or drug addiction, as well as those afflicted with chronic diseases and neonates with abnormalities. Through the implementation of these inclusion and exclusion criteria, the study sought to attain a representative sample while simultaneously minimizing the potential influence of external factors on MHL and neonatal anthropometric factors.

During the post-enrollment phase, pregnant women were subject to a follow-up period until delivery. This phase involved vigilant monitoring aimed at identifying any potential medical complications or alterations arising from their pregnancies. The systematic and routine assessments conducted during these follow-up visits were intended to safeguard the participants' well-being and oversee the favorable advancement of their pregnancies. These visits were scheduled at specified intervals, typically on a monthly or quarterly basis, aligning with established healthcare protocols for expectant mothers.

### Variables and data collection

In our investigation, the primary outcomes included the anthropometric characteristics of neonates, encompassing BW, SL, and HC. These metrics were ascertained within the first 24 h following birth, employing standardized methodologies and equipment for precise recording. The principal exposure variable under scrutiny was MHL, evaluated through the use of a validated questionnaire. Additionally, we considered various predictive factors, including maternal age, educational attainment, socio-economic status, gravidity, gestational age (GA), and others, based on prior literature suggesting their potential link to neonatal anthropometric outcomes. To mitigate the influence of potential confounding variables on the association between MHL and neonatal anthropometric factors, we also accounted for variables such as maternal smoking status, utilization of prenatal care, pre-existing medical conditions, and maternal nutritional status in our statistical analyses. Furthermore, we explored potential effect modifiers, such as maternal age and educational level, to assess whether the relationship between MHL and neonatal anthropometric factors varied across different subgroups. Data collection was comprehensive and encompassed the entire study duration, commencing from the recruitment phase and continuing throughout the follow-up period. Various data collection methods, including questionnaires, medical assessments, anthropometric measurements, and interviews, were employed to obtain a thorough understanding of subjects' health outcomes, experiences, and general well-being.

To mitigate potential biases, various strategies were incorporated into the study. Initially, a cohort study research design was adopted to monitor the participants over time, thereby reducing the likelihood of selection bias. Furthermore, standardized and validated measurement methodologies were utilized to evaluate both neonatal anthropometric factors and MHL. The personnel involved in data collection underwent training to ensure uniformity and diminish observer bias. Additionally, an exhaustive review of existing literature was undertaken to identify potential confounding variables, which were subsequently considered in the analysis to minimize their impact on the observed relationships and fortify the credibility of our results.

### Instruments

The information was collected using a structured checklist designed to document maternal socio-demographic and obstetric characteristics, maternal health-related factors (including MHL, pregnancy outcomes, and happiness), and neonatal physical measurements (including BW, SL, and HC).

#### The socio-demographic checklist

The maternal socio-demographic information, encompassing age, body mass index (BMI), educational attainment, occupation, and geographical location, as well as the corresponding details regarding the age, educational background, and occupation of their spouses, were obtained. Furthermore, obstetric factors including gravidity, medical history, miscarriage history, and stillbirth history were considered. Additionally, neonatal anthropometric parameters such as birth weight (BW), crown-heel length (SL), and head circumference (HC) were assessed. These measurements were derived from medical records, birth registries, or direct measurements conducted shortly after delivery.

#### Maternal health literacy and pregnancy outcomes questionnaire

The Maternal Health Literacy and Pregnancy Outcomes Questionnaire (MHLAPQ) was employed to evaluate participants’ MHL levels and pregnancy outcomes. The MHLAPQ consists of 26 items: 14 items assess maternal health literacy, and 12 items examine pregnancy outcomes (POs). Each item is rated on a five-point Likert scale ranging from “completely agree” to “completely disagree.” The original version of the instrument, developed by Mojoyinola, demonstrated acceptable internal consistency with a Cronbach’s α coefficient of 0.81 [13].

A subsequent validation study by Kharazi et al. on the Persian adaptation of the MHLAPQ yielded improved reliability, with Cronbach’s α coefficients of 0.89 for the health literacy section and 0.67 for the POs section [14]. For the purposes of this study, we utilized the updated Persian version in Semnan. In addition to analysing MHL as a continuous variable, we also considered previously reported categorizations of MHL into insufficient, borderline, and sufficient levels based on established thresholds from prior research [15]. Although these cut-off points provided a framework for contextualizing participants’ MHL distribution, our primary analyses focused on treating MHL scores as a continuous measure.

#### Happiness questionnaire

The happiness questionnaire developed by Abachizadeh et al. (2015) was employed to assess the happiness level of individuals. This tool comprises 44 items designed to be more than the “Oxford University Excellence Assessment Questionnaire.” Some of the items are tailored to the cultural differences between Iranians and other nations; for example, “Sincere communication with God gives me a sense of happiness.” Specifically, it is structured into two sections: the initial segment encompasses 30 items through which the participants express their agreement with components associated with happiness from “strongly agree” to “strongly disagree,” while the subsequent section comprises 14 items enabling respondents to articulate their feelings about happiness-related components, utilizing a Likert scale from “always” to “never” [16].

#### Neonatal anthropometric parameters

The expectant mothers were monitored until childbirth, and the neonatal anthropometric measurements were conducted postpartum. The study utilized flexible measuring tapes and a digital baby weighing scale for data collection, typically carried out within 24 h of birth. Specifically, the newborns' BW was assessed using a specialized digital baby weighing scale for precise measurement, while the SL was recorded with an infantometer, ensuring the infant's supine position and full extension. Additionally, HC measurements were taken by positioning the measuring tape anteriorly at the forehead and posteriorly along the most prominent point at the back of the head [17].

### Data analysis

Data analysis was performed using IBM® SPSS® Statistics software (Version 26.0). Continuous variables were summarized using means and standard deviations, while categorical variables were reported as frequencies and percentages. Differences in mean scores between two groups were evaluated using independent t-tests, and comparisons among more than two groups were conducted using one-way analysis of variance (ANOVA). When ANOVA results were statistically significant, post hoc tests were performed to determine pairwise differences. Associations between continuous variables were assessed using Pearson’s correlation coefficient. A confidence interval of 95% and a significance level of less than 5% (p < 0.05) were applied across all statistical analyses.

## Results

### Summary of maternal and neonatal characteristics

A comprehensive set of variables related to the study is shown in Table 1, encompassing both demographic and obstetric aspects. It includes the mean ± SD for key factors such as age (28 ± 5.67 years), BMI (23.84 ± 4.12), parental age (32.32 ± 5.32), and other variables. Additionally, it provides the frequency distribution for current maternal age (with 57.2% for 20–29 year age group), maternal (80.9% for Diploma/University level) and paternal educational levels (81.6% for Diploma/University level), maternal (69.7% for housewife) and paternal (35.2% for employee) occupation, gestation age (95.6% for > 25 weeks), gravidity (95.6% first pregnancy), history of miscarriage (76.6% for no miscarriage) and stillbirth (94.8% for), as well as neonatal physical measurements at birth like BW (3284.98 ± 1213.96), HC (33.99 ± 3.21), and SL (47.84 ± 6.62). This detailed dataset offers valuable insights into the demographic composition, health profiles, and socio-economic characteristics of the study population, providing a foundation for further analysis and interpretation in the context of maternal and child health, demographic trends, and related research areas.

**Table 1:** Demographic characteristics and Obstetric factors of the pregnant women (n = 591)

Variables	Frequency (%)	Mean ± SD
Age (year)	–	28 ± 5.67
BMI	–	23.84 ± 4.12
Spouse's age (year)	–	32.32 ± 5.32
Current maternal age (year)		
≤ 19	18 (3)	–
20–29	338 (57.2)	–
30–39	230 (38.9)	–
≥ 40	5 (0.8)	–
Educational level		
No formal education	19 (2.90)	–
Primary school	32 (5.4)	–
Secondary School	62 (10.5)	–
Diploma or University Level	478 (80.9)	–
Education status of spouse		
No formal education	33 (5.6)	–
Primary school	21 (3.6)	–
Secondary School	55 (9.3)	–
Diploma or University Level	482 (81.6)	–
Maternal occupation		
Housewife	412 (69.7)	–
Employee	119 (20.1)	–
Daily worker	22 (3.7)	–
Self-employed	38 (6.4)	–
Spouse's occupation		
Unemployed	21 (3.6)	–
Employee	208 (35.2)	–
Daily worker	161 (27.2)	–
Self-employed	201 (34)	–
Gestation Age		
< 12 weeks	0	–
13–24 weeks	26 (4.4)	–
> 25 weeks	565 (95.6)	–
Gravidity		
1	227 (38.4)	–
2	202 (34.2)	–
≥ 3	162 (27.4)	–
Number of deliveries		
0	22 (3.7)	–
1	251 (42.5)	–
2	241 (40.8)	–
≥ 3	77 (13)	–
History of miscarriage		
0	453 (76.6)	–
1	107 (18.1)	–
2	23 (3.9)	–
≥ 3	8 (1.4)	–
History of stillbirth		
0	560 (94.8)	–
1	27 (4.6)	–
2	2 (0.3)	–
≥ 3	2 (0.3)	–
Residence		
Urban	511 (86.4)	–
Rural	80 (13.5)	–
MHL	–	57.34 ± 8.67
PO	–	45.91 ± 7.57
Happiness	–	122.88 ± 25
BW (g)	–	3284.98 ± 1213.96
HC (cm)	–	33.99 ± 3.21
SL (cm)	–	47.84 ± 6.62

*SD* Standard deviation, *MHL* maternal health literacy, *PO* pregnancy outcome, *BW* body weight, *HC* head circumference, *SL* supine length, *g* grams, *cm* centimeters

### Distribution of maternal health literacy levels by socio-demographic and obstetric factors

According to Table 2, sufficient MHL levels were observed more frequently among women 30–39 age group (1.4%), women with a diploma/university degree (2.0%), housewives (1.5%), women in later pregnancy ages (> 25 weeks) (2.5%), and those with no history of miscarriage (1.5%) or stillbirth (1.9%).

**Table 2:** The frequency of maternal health literacy levels based on socio-demographic characteristics and obstetric factors of the pregnant women (n = 591)

Variables	MHL level							
	Inadequate		Not adequately sufficient		Sufficient		Excellent	
	N	%	N	%	N	%	N	%
Maternal age group								
≤ 19	8	1.4	6	1.0	4	0.7	0	0.0
20–29	67	11.3	269	45.5	2	0.3	0	0.0
30–39	44	7.4	172	29.1	8	1.4	6	1.0
≥ 40	0	0.0	5	0.8	0	0.0	0	0.0
Maternal educational level								
No formal education	13	2.2	6	1.0	0	0.0	0	0.0
Primary school	22	3.7	9	1.5	1	0.2	0	0.0
Secondary School	29	5.1	32	5.2	1	0.2	0	0.0
Diploma/University	54	9.1	406	68.7	12	2.0	6	1.0
Husband's educational level								
No formal education	21	3.6	12	2.1	0	0.0	0	0.0
Primary school	14	2.4	6	1.0	1	0.2	0	0.0
Secondary School	23	3.9	32	5.4	0	0.0	0	0.0
Diploma/University	60	10.2	403	68.2	13	2.2	6	1.0
Maternal occupation								
Housewife	101	17.1	298	50.4	9	1.5	4	0.7
Employee	10	1.7	105	17.8	2	0.3	2	0.3
Daily worker	6	1.0	16	2.7	0	0.0	0	0.0
Self-employed	2	0.3	33	5.6	3	0.5	0	0.0
Husband’s occupation								
Unemployed	5	0.8	16	2.7	0	0.0	0	0.0
Employee	23	3.9	177	29.9	11	1.9	0	0.0
Daily worker	59	10.2	100	16.9	2	0.3	0	0.0
Self-employed	31	5.2	160	27.0	1	0.2	6	1.0
Residence								
Urban	96	16.2	404	68.4	7	1.2	4	0.7
Rural	23	3.9	48	8.1	7	1.2	2	0.3
Gestation Age								
< 12 weeks	0	0.0	0	0.0	0	0.0	0	0.0
13–24 weeks	1	0.1	25	4.3	0	0.0	0	0.0
> 25 weeks	118	20.0	425	72	15	2.5	7	1.2
Gravidity								
1	43	7.3	178	30.1	6	1.0	0	0.0
2	44	7.4	156	26.4	0	0.0	2	0.3
3 and more	33	5.6	117	19.8	8	1.4	4	0.7
History of miscarriage								
0	100	16.9	342	57.9	9	1.5	2	0.3
1	14	2.4	85	14.4	4	0.7	4	0.7
2	2	0.3	20	3.4	1	0.2	0	0.0
3 and more	3	0.5	5	0.9	0	0.0	0	0.0
History of stillbirth								
0	112	19.0	431	72.9	11	1.9	6	1.0
1	5	0.8	19	3.2	3	0.5	0	0.0
2	2	0.3	0	0.0	0	0.0	0	0.0
3 and more	0	0.0	2	0.3	0	0.0	0	0.0

*MHL* maternal health literacy

Women in later pregnancy ages (> 25 weeks) had the highest proportion of inadequate MHL (20.0%), while those in the second trimester had the lowest (0.1%). A history of miscarriage or stillbirth was also associated with lower MHL, with the highest proportion of inadequate MHL among those with no history of miscarriage (16.9%) and stillbirth (19.0%), while the lowest was among those with two miscarriages (0.3%) and stillbirths (0.3%). Rural residents with inadequate MHL were 3.9%, compared to 16.2% in urban areas (Table 2).

### Relationship of health literacy, pregnancy outcomes, and happiness with maternal, paternal, and obstetric characteristics

According to Table 1, the mean MHL score was 57.34 ± 8.67. A comparison of the mean MHL scores was conducted based on demographic characteristics and obstetric factors (Table 3). Significant differences were found in the MHL scores based on maternal educational level (p = 0.0001), paternal educational level (p = 0.0001), maternal occupation (p = 0.0001), paternal occupation (p = 0.0001), and history of miscarriage (p = 0.01). Specifically, higher MHL scores were observed in association with maternal education level of diploma/university, paternal education level of diploma/university, maternal occupation as an employee, paternal occupation as an employee, and a history of miscarriage with more than two occurrences.

**Table 3:** Comparing maternal health literacy, pregnancy outcome, and happiness score based on socio-demographic characteristics and obstetric factors of the pregnant women (n = 591)

Variables	MHL		*p*-value	PO		*p*-value	Happiness		*p*-value
	Mean	SD		Mean	SD		Mean	SD	
Current maternal age			0.129			0.075			0.742
≤ 19	51.50	15.83		42.17	10.11		118.78	22.87	
20–29	55.89	11.18		46.17	6.96		123.23	25.59	
30–39	58.03	14.33		45.88	8.07		122.93	24.34	
≥ 40	61.40	4.77		43.40	11.50		111.20	24.73	
Maternal educational level			0.0001			0.0001			0.66
No formal education	34.16	17.22		42	10.95		124.89	22.77	
Primary school	42.91	13.61		41.44	5.55		127.50	19.72	
Secondary School	50.10	11.48		42.82	8.81		121.56	21.59	
Diploma/University	59.30	10.64		46.76	7.10		122.66	25.82	
Spouse's educational level			0.0001			0.0001			0.89
No formal education	39.09	16.33		40.61	7.70		124.42	19.41	
Primary school	43.86	13.75		40.33	6.38		119.95	19.58	
Secondary School	51.36	11.48		43.42	7.04		121.42	23.59	
Diploma/University	59	10.93		46.80	7.37		123.07	25.74	
Maternal occupation			0.0001			0.0001			0.018
Housewife	55	12.80		45.07	7.43		123.29	25.13	
Employee	62	6.79		48.45	7.06		119.30	27.03	
Daily worker	54.27	12.55		47.45	7.61		137.32	20.57	
Self-employed	61.02	12.27		46.16	8.86		121.26	14.60	
Spouse's occupation			0.0001			0.0001			0.106
Unemployed	54.76	12.68		43.95	6.51		124.90	25.03	
Employee	59.89	13.98		47.27	7.94		119.94	26.04	
Daily worker	50.24	13.63		43.25	7.55		122.55	25.24	
Self-employed	58.64	7.96		46.85	6.75		125.97	23.46	
Residence			0.04			0.0001			0.0001
Urban	34.78	8.28		42.32	8.48		125.32	33.93	
Rural	37.18	17.47		43.23	11.52		115.21	27.90	
Gravidity			0.336			0.124			0.11
1	57.44	11.01		46.48	7.66		118.52	24.91	
2	56.63	11.51		46.06	6.83		123.89	23.09	
≥ 3	55.52	15.84		44.91	8.23		127.73	26.51	
History of miscarriage			0.01			0.779			0.761
0	55.72	12.56		45.80	7.29		122.27	24.82	
1	59.62	13.48		46.26	8.91		124.84	24.73	
2	61.13	7.26		46.96	6.34		124.87	30.19	
≥ 3	56.13	13.12		45.38	7.95		125.50	26.15	
History of stillbirth			0.644			0.122			0.365
0	56.59	12.70		46.05	7.57		122.66	24.52	
1	57.81	12.67		44.04	6.76		129.33	33.80	
2	51.50	15.83		44.22	2.43		115.2	22.18	
≥ 3	55.89	11.18		45.50	3.54		118.1	31.11	

*SD* standard deviation, *MHL* maternal health literacy, *PO* pregnancy outcome

According to Table 1, the mean score of PO was 45.91 ± 7.57. A comparison of the mean PO scores was conducted based on demographic characteristics and obstetric factors (Table 3). Significant differences were observed in the PO scores based on maternal educational level (*p* = 0.0001), paternal educational level (*p* = 0.0001), maternal occupation (*p* = 0.0001), and paternal occupation (*p* = 0.01). Specifically, higher PO scores were associated with maternal education level of diploma/university, paternal education level of diploma/university, maternal occupation as an employee, and paternal occupation as an employee.

According to Table 1, the mean score of happiness was 122.88 ± 25. A comparison of the mean happiness scores was conducted based on demographic characteristics and obstetric factors (Table 3). Significant differences were observed in the happiness scores based on maternal occupation (*p* = 0.018) and residency location (*p* = 0.0001). Higher happiness scores were observed in housewives.

### Relationship of neonatal measurements at birth with maternal, paternal, and obstetric characteristics

According to Table 1, the mean neonatal BW was 3284.98 ± 1213.96 g. A comparison of the mean BW was conducted based on demographic characteristics and obstetric factors (Table 4). Significant differences were observed in the neonatal BW based on maternal occupation (*p* = 0.038) and history of stillbirth (*p* = 0.047). Specifically, lower neonatal BW was observed in neonates from mothers with a daily worker occupation and history of more than 3 times of stillbirth.

**Table 4:** Comparing neonatal BW, HC, and SL based on socio-demographic characteristics and obstetric factors of the pregnant women (n = 591)

Variables	BW		*p*-value	HC		*p*-value	SL		*p*-value
	Mean	SD		Mean	SD		Mean	SD	
Current maternal age			0.530			0.878			0.956
≤ 19	2931.89	457.08		34.44	4.86		47.28	7.45	
20–29	3140.08	528.83		33.99	3.41		47.96	6.28	
30–39	3164.30	491.49		33.94	2.77		47.72	7.10	
≥ 40	3716	224.23		34.60	1.82		48.00	4.64	
Maternal educational level			0.161			0.114			0.673
No formal education	3406.32	705.47		32.83	4.21		47.32	8.35	
Primary school	3202.03	596.09		34.42	3.91		46.91	6.11	
Secondary School	3284.21	539.81		33.37	3.61		47.25	8.69	
Diploma/University	3116.50	491.26		34.09	3.05		48.01	6.28	
Spouse's educational level			0.454			0.652			0.282
No formal education	3194.70	671.86		33.47	5.18		46.76	7.99	
Primary school	3155.48	581.56		33.83	1.95		47.38	5.60	
Secondary School	3151.02	576.25		33.68	3.34		46.53	8.41	
Diploma/University	3137.33	490.97		34.07	3.07		48.09	6.32	
Maternal occupation			0.038			0.118			0.045
Housewife	3147.05	509.79		34.14	3.35		48.10	6.72	
Employee	3107.45	484.88		33.87	2.32		47.77	4.90	
Daily worker	2904.82	554.33		33.36	2.66		44.96	9.68	
Self-employed	3374.61	573.80		33.58	3.98		48.50	5.74	
Unemployed			0.665			0.282			0.113
Spouse’s occupation	2980.95	408.39		33.83	2.21		49.10	6.10	
Employee	3119.52	472.09		34.27	3.29		48.28	5.48	
Daily worker	3182.55	604.38		34.07	3.28		48.24	6.74	
Self-employed	3167.36	484.34		33.66	3.16		46.94	7.55	
Gravidity			0.467			0.703			0.066
1	3076.90	521.89		34.11	2.98		48.35	5.92	
2	3173.86	522.53		33.86	3.82		46.97	7.76	
≥ 3	3215.52	481.95		33.99	2.67		48.24	5.91	
History of miscarriage			0.839			0.694			0.577
0	3351.51	539.13		33.97	2.94		47.82	6.70	
1	3089.01	397.45		34.22	4.28		48.33	6.08	
2	3061.96	457.47		33.37	3.15		46.22	8.02	
≥ 3	3113.75	518.76		34.25	1.49		47.50	5.07	
History of stillbirth			0.047			0.072			0.023
0	3262.18	512.02		34.01	3.20		47.90	6.59	
1	33,045	519.71		33.89	2.89		47.63	5.94	
2	2896.07	10.61		28.00	8.49		31.50	9.19	
≥ 3	2692.50	134.35		36.21	4.21		52.75	1.06	

*SD* standard deviation, *BW* body weight, *HC* head circumference, *SL* supine length

According to Table 1, the mean neonatal HC was 33.99 ± 3.21 cm. A comparison of the mean HC scores was conducted based on demographic characteristics and obstetric factors (Table 4). There was no significant difference in the neonatal HC in terms of maternal, paternal, and obstetric characteristics.

According to Table 1, the mean neonatal SL was 47.84 ± 6.62 cm. A comparison of the mean SL scores was conducted based on demographic characteristics and obstetric factors (Table 4). Significant differences were observed in the neonatal SL in terms of maternal occupation (*p* = 0.023). Specifically, a greater SL was observed in neonates from mothers aged over 40, while a smaller SL was observed in neonates from mothers with a daily worker occupation.

### Relationship between neonatal measurements and score of questioners

We conducted a correlation analysis study between neonatal anthropometric measurements and MHL, POs, and happiness scores (Table 5). According to our results, significant correlations were observed between BW and HC (*r* = 0.143 and *p* = 0.0001), SL (*r* = 0.245 and *p* = 0.0001), and MHL (*r* = 0.082 and *p* = 0.0001). In addition, there was a significant correlation between HC and SL (*r* = 0.214 and *p* = 0.0001). For obstetric aspects of the study, there were correlations between PO and MHL (*r* = 0.363 and *p* = 0.0001), as well as happiness scores (*r* = 0.180 and *p* = 0.002).

**Table 5:** Relationship between neonatal measurements and maternal health literacy, pregnancy outcome and happiness (n = 591)

	HC	SL	MHL	PO	Happiness
BW					
r	− 0.143	0.245	− 0.082	− 0.047	− 0.025
*p-*value	0.0001	0.0001	0.046	0.252	0.550
HC					
r	1	0.614	0.0001	0.026	0.066
*p-*value	–	0.0001	0.992	0.522	0.107
SL					
r	–	1	− 0.017	− 0.022	0.057
*p-*value	–	–	0.672	0.595	0.167
MHL					
r	–	–	1	0.363	0.021
*p-*value	–	–	–	0.0001	0.613
PO					
r	–	–	–	1	0.180
*p-*value	–	–	–	–	0.042

*MHL* maternal health literacy, *PO* pregnancy outcome, *BW* body weight, *HC* head circumference, *SL* supine length

### Relationship between health literacy levels and neonatal anthropometric measurements

Table 6 presents the mean neonatal anthropometric indices—BW, HC, and SL—across different health literacy levels of the pregnant women. There was no significant relationship between MHL levels and neonatal BW (p = 0.552) or HC (p = 0.505). However, a significant relationship was observed between MHL levels and neonatal SL (p = 0.04) with higher health literacy correlating with increased SL.

**Table 6:** Relationship between maternal health literacy levels and neonatal anthropometric measurements (n = 591)

Variables	Maternal Health literacy level								*p*-value
	Inadequate		Not adequately sufficient		Sufficient		Excellent		
	Mean	SD	Mean	SD	Mean	SD	Mean	SD	
BW	3055.36	541.83	3427.38	937.35	3105.5	771.29	2985.00	560.35	0.552
SL	46.45	5.51	48.90	4.92	48.57	5.39	53.33	0.52	0.04
HC	34.33	1.97	34.19	2.29	34.64	1.55	35.33	1.03	0.505

*SD* standard deviation, *BW* body weight, *SL* supine length, *HC* head circumference

## Discussion

In a 2-year cohort study, we aimed to explore the relationship between maternal socio-demographic and obstetric characteristics, health-related factors, and neonatal anthropometric outcomes. By addressing MHL, subjective happiness, and PO, the research sought to provide insights into the role of these factors in influencing neonatal health. It found that MHL scores were higher among those with higher education levels, employee occupations, and a history of multiple miscarriages. Additionally, PO scores were positively associated with diploma/university education and employee occupations for both mothers and spouses. Furthermore, higher happiness scores were observed in housewives. The study also revealed significant differences in neonatal measurements based on maternal characteristics, with correlations between pregnancy outcomes and neonatal BW, HC, and SL, as well as MHL and happiness scores.

The main results of the study highlight significant variations in MHL scores, PO scores, and maternal happiness based on demographic and occupational factors. Higher MHL scores were associated with elevated educational levels for both mothers and spouses, employee occupations for both, and a history of multiple miscarriages. This underscores the importance of considering these factors when addressing maternal health literacy and suggests potential opportunities for targeted interventions to enhance MHL and improve maternal and child health outcomes. Similarly, higher PO scores were linked to diploma/university education levels and employee occupations for both mothers and spouses, highlighting the role of parental education and occupation in shaping pregnancy outcomes and supporting maternal health. Furthermore, the research demonstrated connections between the PO score, highlighting the significant influence of MHL on the outcome of pregnancy.

HL skills encompass a range of social and cognitive abilities that assess an individual's capacity and motivation to access, comprehend, and utilize information to promote and maintain good health [8]. These skills and resources reflect people's ability to process health-related information effectively [18]. According to the literature, both MHL and the quality of maternal healthcare contribute to maternal–fetal complications [19]. Notably, heightened MHL levels have been linked to a reduction in pregnancy complications related to blood pressure and diabetes in women, while low birth weight, a risk factor for infant mortality, is strongly associated with prenatal healthcare and can be mitigated through improvements in MHL [20]. Pregnant women with inadequate HL are less likely to properly utilize prescribed drugs, such as folic acid during pregnancy, or participate in prenatal screening at an appropriate gestational age, resulting in longer hospitalization periods [21]. Conversely, pregnant women with adequate MHL levels demonstrated a better understanding of the risks associated with tobacco use during pregnancy [22]. In addition, different levels of MHL were recorded in Iran. Inadequate MHL was reported by Asadi et al. [23]. This research group found a low level of HL among pregnant women referred to health centers in Yazd, Iran [23]. However, contrasting findings were reported by Tavananezhad et al. [24], Ghanbari et al. [25], and Moshki et al. [26], who showed higher levels of MHL among pregnant women in Iran. In comparison to pregnant women from high-income countries, those living in low-income countries tend to have lower MHL levels [27]. Similarly, Tavananezhad et al. (2022) demonstrated that the MHL level in pregnant women was associated with their age [24]. In a study by Moshki et al. (2018), it was reported that HL was linked to education but not associated with other demographic variables [26]. Overall, higher MHL is associated with improved maternal outcomes, emphasizing the need for more educational programs to increase HL in pregnant women. Notably, a previous study conducted in Tehran province found that approximately 30% of pregnant women had insufficient MHL [15]. This aligns with our initial assumptions used for the sample size calculation and underscores that challenges related to maternal health literacy may be prevalent across different regions of Iran, not limited to a single locality. Such consistency highlights the importance of implementing broader, regionally adapted interventions aimed at improving MHL and, consequently, pregnancy and neonatal outcomes.

On the other hand, the analysis of the maternal happiness score revealed notable disparities based on occupation and residency location, indicating the influence of these factors on maternal and family health. Additionally, the results of the study revealed the potential relationship between PO score and happiness score, emphasizing the effects of happiness in mothers on the outcomes of pregnancy. Several studies have discussed the key role of happiness in mother and father before, during, and after giving birth [11, 12]. As a stressful period of life, pregnancy is accompanied by some negative behaviors like alcohol and tobacco consumption that may be associated with maternal and fetus consequences [27]. Similarly, lower levels of happiness during pregnancy are related to more requirements of care services and poor POs [27].

We also monitored neonatal BW, HC, and SL at birth as indicators of neonatal health. The comparison of BW and SL in terms of maternal socio-demographic and obstetric factors revealed significant associations. Specifically, lower neonatal BW was observed in neonates from mothers with a daily worker occupation. In addition, lower BW and SL were observed in neonates from mothers with a history of more than 3 stillbirths. These findings underscore the impact of maternal occupation and history of stillbirth on neonatal health indicators. Limited research has been conducted on the effects of maternal sociodemographic and obstetric factors on neonatal health. Additionally, the effects of MHL and happiness on neonatal outcomes were rarely studied. According to our results, there were significant correlations between MHL and neonatal BW. However, no significant correlations between these factors and PO and happiness were recorded.

There is evidence indicating a link between lower maternal mental health literacy (MHL) and increased mortality rates and inadequate self-care [28]. The results of a systematic review (2021) reported that interventions aimed at increasing levels of MHL may be associated with improved knowledge and outcomes for newborns. However, previous evidence has been limited by inconsistent measurements and outcomes [29]. Additionally, neonates from unhappy mothers often experience poor outcomes, such as prematurity and lower BW [30]. In contrast, happier mothers may exhibit positive health behaviors during pregnancy, such as proper nutrition and physical activity, resulting in favorable neonatal outcomes, including full-term deliveries, normal BW, and early initiation of breastfeeding [31, 32]. Several studies have indicated that HC is a significant indicator of brain development and growth, particularly in the first two years of life [33, 34]. Smaller HC may be linked to learning difficulties and a lower intelligence quotient, IQ [33]. Furthermore, the results of Bouthoorn et al. (2012) showed that smaller HC in the first 6 months of life was associated with lower maternal education. Additionally, HC differences were attributed to birth weight, gestational age, and parental height at 6 months [35]. The study's comprehensive assessment of neonatal health indicators in relation to socio-demographic and obstetric characteristics provides valuable insights into the multifaceted nature of neonatal health. These results can inform routine prenatal care, which could incorporate interventions that address MHL and maternal subjective happiness during pregnancy, with the aim of enhancing women's understanding of health information and improving their psychosocial well-being. Additionally, healthcare teams can collaborate with psychologists, social workers, and community support groups to identify and assist women at higher risk of low MHL or reduced happiness, ensuring timely referrals to psychosocial support services. Such integrative and multidisciplinary approaches may improve not only pregnancy and neonatal outcomes but also the overall well-being and long-term health trajectory of mothers and their children.

### Strengths and limitations

The present study has several limitations that should be acknowledged. First, the use of self-reported tools may have introduced response bias, as participants' individual differences and psychological states at the time of completing the questionnaires could have influenced their answers. Second, the study was conducted in a single center, focusing exclusively on pregnant women attending Amir Al-Momenin Hospital in Semnan. This monocentric design limits the generalizability of the findings to other geographic regions and populations.

Despite these limitations, the study also has significant strengths. It is the first in our country to examine the relationship between maternal health literacy, subjective happiness during pregnancy, and neonatal anthropometric outcomes. The use of a large sample size enhances the statistical reliability of the results. Additionally, the application of a pregnancy-specific health literacy questionnaire ensures that the tools were tailored to the unique needs and experiences of the study population, further supporting the validity of the findings.

## Conclusion

In a comprehensive 2-year cohort study, a study of the associations between maternal socio-demographic and obstetric characteristics, different health-related factors, and neonatal anthropometric measurements showed significant findings. The study highlighted the impact of MHL and happiness on PO and neonatal well-being. According to the results, higher MHL scores were associated with higher maternal and paternal education levels, employee occupations, and a history of multiple miscarriages. Moreover, PO scores were associated with higher maternal and paternal education as well as employee occupations, while happiness scores were notably higher among housewives. The analysis further uncovered substantial variations in neonatal measurements in terms of maternal characteristics. Neonates of mothers with daily worker occupations exhibited lower birth weights and history of multiple stillbirths. In addition, multiple stillbirths affected the result of neonatal SL at birth. Further studies are required to reveal the effects of MHL and what happened on pregnancy outcomes. Taken together, these findings underscore the need for healthcare providers to integrate health literacy interventions, psychosocial support, and resilience-building strategies into standard prenatal care. By enhancing both maternal comprehension of health information and emotional well-being, practitioners can contribute to healthier pregnancies, improved neonatal outcomes, and sustained benefits for families and communities.

## Supplementary Information

Below is the link to the electronic supplementary material.

(DOCX 24 kb)

## Data Availability

The data that support the findings of this study are available, but restrictions apply to the availability of these data and so are not publicly available. The data are, however, available from the corresponding authors upon reasonable request and with the permission of Semnan University of Medical Sciences. We confirm that all methods were carried out in accordance with relevant guidelines and regulations.
