# Supplementary material for: Impact of health literacy and subjective happiness in pregnancy on neonatal anthropometry: a cohort study
Source: Discov Ment Health. 2025 Apr 24;5(1):60. doi: 10.1007/s44192-025-00192-8 (PMC12021744; doi:10.1007/s44192-025-00192-8)
Supplement: Supplementary file 1 — (DOCX 24 kb) [file 44192_2025_192_MOESM1_ESM.docx]

**Health Literacy Assessment Questionnaire**

|  | | Strongly Disagree | Disagree | Neutral | Agree | Strongly Agree |
| --- | --- | --- | --- | --- | --- | --- |
| 1 | I can read and write. | 1 | 2 | 3 | 4 | 5 |
| 2 | I can perform basic math operations (e.g., calculating the amount of fruit, vegetables, or meat based on the required portions during pregnancy). | 1 | 2 | 3 | 4 | 5 |
| 3 | I can understand basic medical terms during pregnancy (e.g., anemia, swelling, high blood pressure, etc.). | 1 | 2 | 3 | 4 | 5 |
| 4 | I can correctly read and understand health information. | 1 | 2 | 3 | 4 | 5 |
| 5 | I can understand and interpret basic health information (e.g., brushing teeth, washing hands, etc.). | 1 | 2 | 3 | 4 | 5 |
| 6 | I can easily read, understand, and act according to healthcare instructions. | 1 | 2 | 3 | 4 | 5 |
| 7 | I have enough information and awareness about the required diet during pregnancy and after childbirth. | 1 | 2 | 3 | 4 | 5 |
| 8 | I have sufficient skills to prepare and use a proper food. | 1 | 2 | 3 | 4 | 5 |
| 9 | I can read health pamphlets and acquire skills related to personal and food hygiene during pregnancy and after childbirth. | 1 | 2 | 3 | 4 | 5 |
| 10 | I have limited knowledge and skills regarding infant care after birth (e.g., breastfeeding, bathing, etc.). | 1 | 2 | 3 | 4 | 5 |
| 11 | I have the ability to obtain health information through libraries or the internet. | 1 | 2 | 3 | 4 | 5 |
| 12 | I can read and understand warning signs during pregnancy (e.g., anemia, paleness, shortness of breath, high blood pressure, swelling, bleeding, preterm labor, etc.). | 1 | 2 | 3 | 4 | 5 |
| 13 | I can accurately read, understand, and interpret prescriptions and medical instructions. | 1 | 2 | 3 | 4 | 5 |
| 14 | I can read and understand my next appointment date (e.g., vaccination day, health check-up, medical examinations, etc.). | 1 | 2 | 3 | 4 | 5 |

**Pregnancy Outcome Assessment Questionnaire**

|  | | **Strongly Disagree** | **Disagree** | **Neutral** | **Agree** | **Strongly Agree** |
| --- | --- | --- | --- | --- | --- | --- |
| 1 | If I encounter any warning signs during pregnancy, I seek assistance from a health center or a private physician for appropriate management. | 1 | 2 | 3 | 4 | 5 |
| 2 | I have enough information about warning signs during pregnancy. | 1 | 2 | 3 | 4 | 5 |
| 3 | I regularly participate in prenatal education classes. | 1 | 2 | 3 | 4 | 5 |
| 4 | I possess adequate knowledge regarding vaccinations during pregnancy. | 1 | 2 | 3 | 4 | 5 |
| 5 | I am able to take appropriate action if I encounter any warning signs during pregnancy. | 1 | 2 | 3 | 4 | 5 |
| 6 | I am capable of identifying warning signs during pregnancy. | 1 | 2 | 3 | 4 | 5 |
| 7 | I have received prenatal care in a timely manner. | 1 | 2 | 3 | 4 | 5 |
| 8 | I did not experience stillbirth during this pregnancy. | 1 | 2 | 3 | 4 | 5 |
| 9 | I had a timely delivery. | 1 | 2 | 3 | 4 | 5 |
| 10 | I experienced a safe and comfortable delivery. | 1 | 2 | 3 | 4 | 5 |
| 11 | I encountered numerous complications during my pregnancy. | 1 | 2 | 3 | 4 | 5 |
| 12 | I delivered an infant with a normal birth weight (above 2500 grams). | 1 | 2 | 3 | 4 | 5 |

**Happiness Questionnaire**

| No. | Statement | Level of Agreement | | | | | Neutral |
| --- | --- | --- | --- | --- | --- | --- | --- |
|  |  | Very Much | Much | Average | Little | Very little |  |
| 1 | I feel that I am physically healthy. | 1 | 2 | 3 | 4 | 5 | 6 |
| 2 | I am satisfied with my family life. | 1 | 2 | 3 | 4 | 5 | 6 |
| 3 | I cope well with life’s circumstances. | 1 | 2 | 3 | 4 | 5 | 6 |
| 4 | I feel optimistic about my future. | 1 | 2 | 3 | 4 | 5 | 6 |
| 5 | If I could live again, I would choose the same life path. | 1 | 2 | 3 | 4 | 5 | 6 |
| 6 | I have a good relationship with my family members. | 1 | 2 | 3 | 4 | 5 | 6 |
| 7 | A close spiritual connection provides me with a sense of well-being. | 1 | 2 | 3 | 4 | 5 | 6 |
| 8 | I feel that I am mentally healthy. | 1 | 2 | 3 | 4 | 5 | 6 |
| 9 | There is wisdom in every event that happens to me, whether good or bad. | 1 | 2 | 3 | 4 | 5 | 6 |
| 10 | I pursue clearly defined goals in life. | 1 | 2 | 3 | 4 | 5 | 6 |
| 11 | I am an optimistic person. | 1 | 2 | 3 | 4 | 5 | 6 |
| 12 | My living environment is a good place to live. | 1 | 2 | 3 | 4 | 5 | 6 |
| 13 | I frequently experience physical pain. | 1 | 2 | 3 | 4 | 5 | 6 |
| 14 | Life pressures negatively impact me. | 1 | 2 | 3 | 4 | 5 | 6 |
| 15 | I have good relationships with people around me, aside from family. | 1 | 2 | 3 | 4 | 5 | 6 |
| 16 | I am satisfied with my work. | 1 | 2 | 3 | 4 | 5 | 6 |
| 17 | I have been useful to others. | 1 | 2 | 3 | 4 | 5 | 6 |
| 18 | My life is full of positive feelings. | 1 | 2 | 3 | 4 | 5 | 6 |
| 19 | I think I am considered attractive by others. | 1 | 2 | 3 | 4 | 5 | 6 |
| 20 | I have sufficient financial resources to support my life. | 1 | 2 | 3 | 4 | 5 | 6 |
| 21 | I feel supported by others. | 1 | 2 | 3 | 4 | 5 | 6 |
| 22 | I feel safe where I live. | 1 | 2 | 3 | 4 | 5 | 6 |
| 23 | The society I live in is a good place to live. | 1 | 2 | 3 | 4 | 5 | 6 |
| 24 | I have many things in my life to be grateful for. | 1 | 2 | 3 | 4 | 5 | 6 |
| 25 | I have achieved my desires in life. | 1 | 2 | 3 | 4 | 5 | 6 |
| 26 | I feel I am below my peers. | 1 | 2 | 3 | 4 | 5 | 6 |
| No. | Statement | Level of Agreement | | | | | Neutral |
|  |  | Very Much | Much | Average | Little | Very Little |  |
| 27 | I feel discriminated against in society. | 1 | 2 | 3 | 4 | 5 | 6 |
| 28 | Overall, I am satisfied with my life. | 1 | 2 | 3 | 4 | 5 | 6 |
| 29 | My life is full of negative feelings. | 1 | 2 | 3 | 4 | 5 | 6 |
| 30 | Overall, I consider myself a happy person. | 1 | 2 | 3 | 4 | 5 | 6 |

The next section:

| No. | Statement | Always | Often | Sometimes | Rare | Never | Neutral |
| --- | --- | --- | --- | --- | --- | --- | --- |
| 31 | I have control over my behaviors and emotions. | 1 | 2 | 3 | 4 | 5 | 6 |
| 32 | I have high self-confidence. | 1 | 2 | 3 | 4 | 5 | 6 |
| 33 | I am a sad person. | 1 | 2 | 3 | 4 | 5 | 6 |
| 34 | I feel I have enough energy for my daily activities. | 1 | 2 | 3 | 4 | 5 | 6 |
| 35 | I enjoy my daily activities. | 1 | 2 | 3 | 4 | 5 | 6 |
| 36 | I am an anxious person. | 1 | 2 | 3 | 4 | 5 | 6 |
| 37 | I sleep well. | 1 | 2 | 3 | 4 | 5 | 6 |
| 38 | I feel lethargic and lazy. | 1 | 2 | 3 | 4 | 5 | 6 |
| 39 | I feel calm. | 1 | 2 | 3 | 4 | 5 | 6 |
| 40 | I am mentally alert. | 1 | 2 | 3 | 4 | 5 | 6 |
| 41 | I laugh a lot. | 1 | 2 | 3 | 4 | 5 | 6 |
| 42 | \|  \| \| --- \|  \| I get angry easily. \| \| --- \| | 1 | 2 | 3 | 4 | 5 | 6 |
| 43 | I engage in artistic and creative activities. | 1 | 2 | 3 | 4 | 5 | 6 |
| 44 | I consider myself to have a good sense of humor. | 1 | 2 | 3 | 4 | 5 | 6 |
